# Individual deprivation, regional deprivation, and risk for oral clefts in Argentina

**DOI:** 10.26633/RPSP.2017.110

**Published:** 2017-10-12

**Authors:** Mariela Soledad Pawluk, Hebe Campaña, Monica Rittler, Fernando Adrián Poletta, Viviana R. Cosentino, Juan Antonio Gili, Lucas Gabriel Gimenez, Jorge Santiago López Camelo

**Affiliations:** 1 Estudio Colaborativo Latinoamericano de Malformaciones Congénitas (ECLAMC), Laboratorio de Epidemiología Genética Genética, Centro de Educación Médica e Investigaciones Clínicas (CEMIC-CONICET) Buenos Aires Argentina Estudio Colaborativo Latinoamericano de Malformaciones Congénitas (ECLAMC), Laboratorio de Epidemiología Genética, Centro de Educación Médica e Investigaciones Clínicas (CEMIC-CONICET), Buenos Aires, Argentina.; 2 Estudio Colaborativo Latinoamericano de Malformaciones Congénitas (ECLAMC) Hospital Materno Infantil Ramón Sarda Buenos Aires Argentina Estudio Colaborativo Latinoamericano de Malformaciones Congénitas (ECLAMC), Hospital Materno Infantil Ramón Sarda, Buenos Aires, Argentina.

**Keywords:** Cleft lip, cleft palate, social class, Argentina, Labio leporino, fisura del paladar, clase social, Argentina, Fenda labial, fissura palatina, classe social, Argentina

## Abstract

**Objective.:**

*The aim of this study was to analyze the effects of individual low socioeconomic status (SES) and deprived geographical area (GA) on the occurrence of isolated cleft lip with or without cleft palate (CL±P) in Argentina*.

**Methods.:**

*This case-control study included 577 newborns with isolated CL±P and 13 344 healthy controls, born between 1992 and 2001, from a total population of 546 129 births in 39 hospitals in Argentina. Census data on unsatisfied basic needs were used to establish the degree of geographical area deprivation. An SES index for each individual was established, using maternal age, gravidity, low paternal and maternal education, and low-level paternal occupation. Logistic regression was used to assess the effects of low SES and of deprived GA on CL±P*.

**Results.:**

*A slightly increased risk of CL±P was observed in mothers with a low SES, while a deprived GA showed no effect. Native ancestry, acute maternal illnesses, and poor prenatal care were significant risk factors for CL±P for the mothers with low SES, after using propensity scores to adjust for the demographic characteristics in cases and controls*.

**Conclusions.:**

*Low individual SES slightly increased the risk for CL±P, but a deprived GA did not have that effect. There was no interaction between individual SES and deprived GA. Factors related to low individual SES—including poor prenatal care, low parental education, lack of information, and lifestyle factors—should be primarily targeted as risk factors for CL±P rather than factors related to a deprived place of residence*.

## RESUMEN

**Objetivo.:**

Analizar los efectos de un bajo nivel socioeconómico individual y una zona geográfica desfavorable en la aparición del labio leporino aislado con o sin paladar hendido (LL ± P) en Argentina.

**Métodos.:**

En este estudio de casos y controles se incluyeron 577 recién nacidos con LL ± P aislado y 13 344 controles sanos nacidos entre 1992 y 2001, de un total de 546 129 nacimientos ocurridos en 39 hospitales de Argentina. Para identificar las zonas geográficas desfavorables se utilizaron datos del Índice de Necesidades Básicas Insatisfechas. Se calculó un índice de nivel socioeconómico para cada participante usando la edad materna, el número de embarazos, el nivel de instrucción bajo del padre y la madre, y el nivel de ocupación bajo del padre. Se usó regresión logística para evaluar los efectos de un bajo nivel socioeconómico y una zona geográfica desfavorable en la ocurrencia de LL ± P.

**Resultados.:**

Se observó un riesgo levemente mayor de LL ± P en madres con bajo nivel socioeconómico, mientras que una zona geográfica desfavorable no mostró ningún efecto. La ascendencia indígena, las enfermedades agudas maternas y una atención prenatal deficiente fueron factores de riesgo significativos para LL ± P en madres con bajo nivel socioeconómico, después de ajustar las características demográficas de casos y controles mediante análisis de propensión.

**Conclusiones.:**

Un bajo nivel socioeconómico aumentó levemente el riesgo de LL ± P, pero una zona geográfica desfavorable no mostró ese efecto. No hubo interacción entre un bajo nivel socioeconómico individual y una zona geográfica desfavorable. Los factores relacionados con un bajo nivel socioeconómico individual —inclusive una atención prenatal deficiente, la baja educación de los padres, la falta de información y el estilo de vida— deben abordarse principalmente como factores de riesgo de LL ± P más que los factores relacionados con una zona de residencia desfavorable.

## RESUMO

**Objetivo.:**

Examinar os efeitos do baixo nível socioeconômico individual e área geográfica em situação de carência na ocorrência de fissura labial isolada com ou sem fissura palatina (FL ± P) na Argentina.

**Métodos.:**

Estudo de caso-controle que compreendeu 577 recém-nascidos com FL isolada ± P e 13 344 controles saudáveis, nascidos entre 1992 e 2001, de uma população total de 546 129 nascimentos em 39 hospitais na Argentina. Foram usados dados censitários sobre necessidades básicas existentes para estabelecer o grau de carência das áreas geográficas. Foi determinado um índice de nível socioeconômico para cada indivíduo baseado na idade materna, número de gestações, baixa escolaridade materna e paterna e ocupação paterna de baixo nível. Foi realizada uma regressão logística para avaliar os efeitos do baixo nível socioeconômico e área geográfica em situação de carência na ocorrência de FL ± P.

**Resultados.:**

Observou-se um risco discretamente aumentado de FL ± P em mães com baixo nível socioeconômico, mas nenhum efeito foi verificado quanto à área geográfica em situação de carência. Descendência indígena, doença materna aguda e assistência pré-natal precária foram fatores de risco importantes para FL ± P nas mães com baixo nível socioeconômico, após o uso de escores de propensão para ajustar as características demográficas em casos e controles.

**Conclusões.:**

O baixo nível socioeconômico individual foi associado a um discreto aumento do risco de FL ± P, mas este efeito não foi observado para área geográfica em situação de carência. Não houve interação entre nível socioeconômico individual e área geográfica em situação de carência. Fatores relacionados ao baixo nível socioeconômico individual, como assistência pré-natal precária, baixa escolaridade dos pais, falta de informação e fatores relacionados aos hábitos de vida, devem ser o foco principal porque eles são os fatores de risco para FL ± P, não fatores relacionados ao domicílio em área carente.

Cleft lip with or without cleft palate (CL±P) is among the most prevalent congenital anomalies. In Argentina, for example, it affects approximately 1 in 1 000 newborns (1). A wide variety of studies have attempted to identify the impact of socioeconomic status (SES) on adverse outcomes, particularly perinatal ones. Some studies have suggested that low SES is associated with an increased risk for neural tube defects (2, 3). For orofacial clefts, some studies have found a similar increase in risk (4 7), but other research has not (8 10). Using the Carstairs and Morris deprivation index (11), Clark et al. (12) showed an increased frequency of oral clefts in neighborhoods with the highest level of deprivation. Other researchers have found an association between low maternal SES and the rate of orofacial clefts (13).

Poletta et al. (14) identified two CL±P clusters coinciding with deprived geographical areas (GAs) in Argentina. This finding could be related to typical conditions found in poor regions, including certain environmental factors (pesticides, poor water quality, dietary deficiencies, environmental pollution); low individual SES; and a high rate of Amerindian ancestry. Together with descendants of Europeans, persons of Amerindian heritage comprise the majority of Argentinians. A greater genetic susceptibility for CL±P and a low socioeconomic status are both recognizably associated with Amerindian ancestry (15, 16).

The objective of this study was to assess the risk for isolated CL±P in infants of mothers of low SES who live in a deprived GA, by quantifying the effect of both of those characteristics. The time period that we selected for our research, 1992 through 2001, was based on the fact that Argentina has had critical economic fluctuations over the last two decades. This included a recession from 1995 to 2002, when unemployment and poverty rates increased greatly and the currency was devalued (17).

The health conditions of people living in deprived GAs may be influenced by such risk factors as limited access to health services and medication, insufficient medical equipment and training of health workers, prevailing attitudes towards health, lack of social support, and deficient public health policy (18). Particularly for CL±P, each risk factor could contribute in a direct or indirect way through variables mediating or interacting among these risk factors. Identifying and quantifying the contribution of low SES and of deprived GA could provide health authorities with resources for primary prevention of CL±P and other adverse outcomes.

## MATERIALS AND METHODS

For our work, we used a case-control study design to assess the odds ratios of deprived GAs and low SES for isolated CL±P.

The data for our study came from information on Argentina compiled by the Latin American Collaborative Study of Congenital Malformations (ECLAMC). ECLAMC is a hospital-based registry of congenital malformations in South America. It involves the voluntary collaboration of health professionals (mostly pediatricians) within a network of maternity hospitals. Those professionals register data on over 50 risk factors and demographic characteristics, previous birth outcomes, and prenatal factors, by obtaining information from medical records and by interviewing the mother of a malformed infant and the mother of a healthy control (a nonmalformed infant that is born immediately after each affected newborn and that is paired by sex), before their discharge. Detailed descriptions of the ECLAMC registry and its methodology have been previously published (19).

Following the standard ECLAMC procedures, the demographic data and informed consent for our study were obtained by health professionals through interviews with the mothers before discharge (19).

Our study was approved by the Centro de Educación Médica e Investigaciones Clínica (CEMIC) Institutional Review Board (Office for Human Research Protection, U.S. Department of Health and Human Services, IRB00001745-IORG 0001315).

In Argentina, during the period of 1992 through 2001, a total of 13 344 malformed newborns were registered by ECLAMC in 546 129 births occurring in 39 hospitals. From those 13 344 malformed children, we selected out 850 live and stillborn infants with CL±P. After we excluded stillborn babies and those with multiple anomalies or syndromes, 577 live-borns with isolated CL±P remained. For our analysis, we used data on 13 344 healthy controls born during that same time period.

### Geographic deprivation level

Our sample was recruited from 39 hospitals of Argentina and may not necessarily be representative of the entire population. Representativeness varies in the country because of differences in how many departments are represented in each sample. (The provinces of Argentina are divided into departments (*departamentos*), except for the province of Buenos Aires, which is divided into *partidos*. In this article, the word “department(s)” will be used as the general term unless referring specifically to just the province of Buenos Aires.)

While generalizability is less certain at the country level, it is rather high at the city level. Many ECLAMC hospitals are considered large community hospitals that together cover a relatively large percentage of births in their respective department. According to national census data for Argentina, in 2001, the country had a total population of 36 260 130, and there were 683 495 live births (20). Our analytical sample is obviously a small fraction of the total number of births in these hospitals; nonetheless, this indicates that the ECLAMC hospitals from which our sample is selected are major providers of maternity care in their communities. The representativeness ranges from as high as 97% (as in department of San Miguel de Tucumán) to as low as 10% (as in the *partido* of Lomas de Zamora, in the province of Buenos Aires) (Appendix I).

To identify deprived GAs, we used national census data on unsatisfied basic needs (UBN) (21) of the same period (1992-2001), for the 25 departments in Argentina where the 39 ECLAMC participating maternity hospitals are located, represented by their geographical coordinates (latitude and longitude). The UBN index data were obtained from the 2001 National Population, Household, and Housing Census (20). This national index is used to measure poverty by expressing the percentage of households with unsatisfied basic needs in each department. Its indicators are directly related to four areas of basic needs (housing, health services, basic education, and minimum income), and it comprises four dimensions. The basic needs of each dimension are considered unsatisfied if any one of the following criteria is fulfilled:

- Dwelling quality: 1: Housing: dwelling is in bad conditions in terms of quality and condition of housing materials; 2: Crowding: More than three residents per bedroom.
- Water and sewage: 1: The dwelling is not connected to the public water network; 2: The dwelling is not connected to the public sewer system.
- Childhood education: Children aged between 6 and 12 years do not attend school.
- Subsistence capacity: 1: Four or more people residing in the dwelling per working person; 2: Completed education of the head of the household is two or fewer years of primary school.

We used a Kuldorff spatial scan statistic under the Poisson model (22) to determine geographical areas with statistically significant low or high UBN index values as compared to the mean value of the total Argentinian population. Using the precise location of each hospital as defined by its geographical coordinates (latitude and longitude), this analysis tests a circular area centered at each point, and each point represents 1 of the 39 maternity hospitals (i.e., unit of analysis) in the ECLAMC network. (The maternal home address could not be used, since this variable was not specified in more than 50% of the sample. Therefore, the hospital of birth was used as a proxy for maternal residence.)

The null hypothesis states that the UBN index is homogeneous in the whole sample, and the alternative assumes deprivation or nondeprivation within a given area, as compared with the UBN index observed outside that area. This test uses the maximum likelihood ratio to determine the areas with the smallest probability for the observed unusual UBN index. The *P* value was obtained through multiple simulations by the Monte Carlo model of 999 replications. Cluster regions were not established a priori. Our analysis had two conditions: the radius of the cluster had to be smaller than 500 km, and the resulting areas should not overlap.

### Individual-level socioeconomic status

A confirmatory factor analysis was performed to establish the latent variable SES as a proxy for individual SES. This variable was composed of seven directly observed, binomial variables: 1) maternal age ≤ 19 years; 2) maternal age ≥ 35 years; 3) maternal primigravidity; 4) multigravidity (three or more pregnancies); 5) low paternal education (from no schooling to incomplete grammar school); 6) low maternal education (also from no schooling to incomplete grammar school); and 7) low-level paternal occupation (unemployed, househusband, or odd job/unskilled labor).

We used LISREL 8.80 software (Scientific Software International, Inc., Chicago, Illinois, United States of America) for structural equation modeling to evaluate different models. The model that best adjusted was: low maternal age, multigravidity, low paternal and maternal education, and low-level paternal occupation. Scores were estimated for each newborn’s family. The 75th percentile was used to classify families, creating two groups: a) families of low SES and b) families of medium or high SES. For simplicity, the second group was called families “of not-low SES.”

The following variables, based on maternal self-reports, were analyzed as possible confounders: few prenatal visits (fewer than five); acute and chronic maternal illnesses; maternal medication (any medicine use during the first trimester of pregnancy (yes/no)); and “native ancestry.” (“Native ancestry” was defined as the lack of recognized ancestors from outside Latin America. That is, the mother only knew about specific child ancestors who were born in Latin America, as far back as she could remember (generally up to great great-grandparents). As measured, native-only ancestry does not necessarily mean indigenous ancestry. Indeed, for the majority of children, native-only ancestry indicates that all the child’s ancestors whom the mother can remember were born in Latin America.)

### Statistical analyses

A Poisson regression was used to estimate the CL±P frequency, according to geographical deprivation level. Logistic regression, using information on mothers of control newborns, was carried out to assess the risk factors in deprived GAs. The GA of maternal residence (deprived GA or not-deprived GA) was the dependent variable, while the independent variables were the confounders defined above.

Odds ratios (ORs) and 95% confidential intervals (CIs) were used to estimate the risk of low SES in deprived areas and in not-deprived areas. ORs were compared with a Mantel-Haenszel test, to evaluate any interaction between risk factors and deprived GA.

Logistic regression was used to assess the adjusted effects of low SES in deprived and not-deprived areas.

Propensity scores were calculated in order to balance the demographic characteristics of cases and controls, thereby assessing the residual effect of low SES in deprived GAs (Appendix II). The propensity scores were incorporated in the model as dummy variables. The following logistic regression model was evaluated:

$$P\left(\frac{Y}{x}\right)=\frac{1}{e^{-a1LowSES+a2GeoDep+\sum a3iXi+\sum a4ibi+\sum a5i\mathrm{year}i}}$$

In this model, a1 is the coefficient of low SES mothers; a2 is the coefficient of deprived geographic areas; a3_i_ is the coefficients of Xi risk factors; a4_i_ b_i_ is the coefficients of dummy variables propensity scores; and a5_i_ is the coefficients of dummy variables of year_i_, for each year of the 1992-2001 period.

The sample size was calculated based on a 20% low-SES prevalence. The 577 cases and 13 344 controls used in this study made it possible to identify a minimum OR of 1.50, for an 80% power of the test (β = 0.20) and a type I error of α < 0.05.

## RESULTS

### Cluster analysis

The Kuldorff analysis identified three geographic clusters with significantly high UBN index values (GAs 1, 2, and 3) and one GA with a significantly low UBN index value (GA 4) (Table 1). Cluster 5 represented the remaining departments, with a medium UBN index value.

The three clusters with high UBN index values were then grouped into a single cluster called “deprived GAs,” while the two clusters with low UBN index values (Cluster 4 and Cluster 5) were grouped into another cluster, called “not-deprived GAs.”

The frequency of CL±P was 8.2 per 10 000 births (95% CI: 6.5, 10.2) in not-deprived GAs and 12.3 per 10 000 (95% CI: 10.8, 13.9) in deprived GAs, where it increased with increasing GA deprivation (incidence rate ratio = 1.28, *P* = 0.023). The CL±P prevalence of the three clusters of deprived GAs was significantly higher than that of the remaining departments (GA 5).

### Individual socioeconomic status

The factor analysis for the construction of individual SES values, including young maternal age, multigravidity, low maternal and paternal education, and low-level paternal occupation, had a goodness of fit of χ^2^ 1 df = 0.11, *P* = 0.739. The residual between observed and expected data had a root mean square error of approximation (RMSEA) of 0.022 (95% CI: 0.011, 0.036). According to the distribution of the SES scores, the mothers were divided into those of low SES (≥ 75^th^ percentile) and those of not-low SES.

Among the demographic characteristics more common among those living in deprived geographical areas were low SES, native ancestry, maternal acute illnesses, and fewer prenatal visits. On the other hand, their frequencies of chronic diseases and of first trimester medication were lower (Table 2).

**TABLE 1.** Geographical areas (GAs) by location, number of hospitals, population, unsatisfied basic needs (UBN) index, and rate (per 10 000 births) of cleft lip with or without cleft palate (CL±P), with 95% confidence interval (CI), Argentina, 1992–2001

GA	Name	Department(s)	Latitude	Longitude	No. of hospitals	Population of GA	UBN (%)	Births	No. of CL±P	CL±P rate	95% CI
1	South	Bariloche	41 58 00 S	71 32 00 O	1	143 415	20.6	6 632	12	18.1	9.4-31.6
		Futaleufu	42 54 49 S	71 18 39 O	1						
2	Buenos Aires Suburban	Esteban Echeverria	34 49 9 S	58 27 57 O	1	1 343 797	18.6	64 454	80	12.4	9.8-15.4
		Lomas de Zamora	34 45 51 S	58 25 44 O	1						
		Almirante Brown	34 50 24 S	58 23 34 O	2						
3	North-West	Manuel Belgrano	24 11 13 S	65 17 57 O	2	905 315	18.4	132 266	158	11.9	10.2-14.0
		San Miguel de Tucumán	26 49 50 S	65 12 13 O	1						
		La Rioja	29 24 47 S	66 51 21 O	1						
4	Buenos Aires City	Buenos Aires	34 36 30 S	58 22 19 O	5	2 725 488	7.8	97 408	80	8.2	6.5-10.2
5	Rest of Argentina	16	NA^a^	NA	24	6 113 640	13.0	245 369	245	10.1	8.8-11.4
Total	Argentina total	25	NA	NA	39	11 231 655	17.7	546 129	577	10.5	9.7-11.4

***Source:*** Prepared by the authors from the study data.

a NA = not applicable.

**TABLE 2.** Demographic characteristics, with 95% confidence intervals (CIs), in deprived geographic areas (GAs) and not-deprived GAs of Argentina, 1992-2001

Risk factor	Deprived GAs (*N* = 11 106)			Not-deprived GAs (*N* = 2 338)		
	N	%	95% CI^a^	N	%	95% CI
Maternal age ≤ 19	2 336	21.2	20.3–21.8	367	15.7	14.2–17.2
Maternal age ≥ 35	1 247	11.3	10.6–11.8	268	11.5	10.2–12.8
Primigravidity	2 939	26.7	25.6–27.3	855	36.6	34.6–38.6
Multigravidity > 3	3 878	34.9	34.0–35.8	497	21.3	19.6–23.0
Low maternal education	2 272	20.6	19.7–21.2	166	7.1	6.1–8.2
Low paternal education	2 089	19.0	18.1–19.5	120	5.1	4.3–6.1
Low-level paternal occupation	4 700	42.3	41.4–43.2	619	26.5	24.7–28.3
Low socioeconomic status.	7 797	70.8	69.3–71.1	1004	42.9	40.9–45.0
Native ancestry	9 603	87.3	85.8–87.1	1916	82.0	80.3–83.5
Maternal acute illness	3 187	29.0	27.9–29.5	530	22.7	21.0–24.4
Maternal chronic disease	1 310	11.9	11.2–12.4	383	16.4	14.9–17.9
Maternal medication	4 975	45.2	43.9–45.7	1141	48.8	46.8–50.8
Prenatal visits < 5	2 813	25.6	24.5–26.1	252	10.8	9.5–12.1

***Source:*** Prepared by the authors from the study data.

### Low socioeconomic status and other risk factors for cleft lip with or without cleft palate

Table 3 shows the number of cases and controls and the frequency of exposure to risk factors in the deprived areas and in the not-deprived areas. The univariate analysis showed that low SES, native ancestry, acute and chronic maternal illnesses, maternal medication, and few prenatal visits were risk factors for CL±P in deprived GAs. In contrast, in not-deprived GAs, only three variables were of risk: maternal acute illnesses, maternal medication, and few prenatal visits. There were no significant OR differences between the deprived GAs and the not-deprived GAs, thereby excluding any interaction between risk factors and GA deprivation (Table 3).

Five propensity score strata were defined, and their distribution is shown in Appendix II. After incorporating propensity scores to adjust for SES as well as years as dummy variables in the model, a slight residual risk for CL±P remained from low SES and native ancestry, while maternal acute illnesses and few prenatal visits showed significant odds ratios. No effect of GA deprivation was observed (Table 4).

### DISCUSION

Since the objective of the study was to analyze the impact of poverty on CL±P, we selected the 1992-2001 period to study the births occurring in Argentina. Argentina suffered an economic downturn that began in late 1998 and intensified in 2001 and 2002, with Argentina’s gross domestic product declining and the unemployment rate increasing (17).

Two poverty indicators were used in this study: 1) a national population index of unsatisfied basic needs (UBN), to evaluate adverse social conditions in Argentina related to maternal residence, and 2) socioeconomic status (SES), to assess poverty at an individual level. In order to evaluate unmeasured variables related to SES, we created an index that fitted the observed correlation matrix. This index included variables related to SES in South America, such as parental age and education, gravidity, and paternal occupation (23). The use of an index to measure SES provides statistical efficiency and a simple presentation of results. In contrast, several single measurements “may lead to collinearity and cluttered results, especially when the intention is to reflect a single significant concept, such as SES, rather than examining the unique contribution of each component,” according to Wehby and colleagues (17).

**TABLE 3.** Univariate analysis of risk factors for cleft lip with or without cleft palate (CL±P) in deprived and not-deprived geographic areas (GAs) of Argentina, 1992-2001

Risk factor	Deprived GAs						Not-deprived GAs						Heterogeneity	
	Cases (*N* = 497)		Controls (*N* = 11 006)		OR	P	Cases (*N* = 80)		Controls (*N* = 2 338)		OR	P		
	Exposed	%	Exposed	%			Exposed	%	Exposed	%			χ^2^ (1 df)	P
Low socioeconomic status	375	75.5	7 797	70.8	1.27	0.027	39	48.8	1 004	42.9	1.26	0.302	0.01	0.991
Native ancestry	441	88.7	9 603	87.3	1.52	0.014	63	78.8	1 916	82.0	0.91	0.740	2.35	0.126
Maternal acute illness	175	35.2	3 187	29.0	1.40	0.001	30	37.5	530	22.7	2.09	0.002	2.45	0.117
Maternal chronic disease	70	14.1	1 310	11.9	1.26	0.076	15	18.8	383	16.4	1.19	0.544	0.03	0.857
Maternal medication	249	50.1	4 975	45.2	1.29	0.006	49	61.3	1 141	48.8	1.71	0.022	1.22	0.269
Prenatal visits < 5	173	34.8	2 813	25.6	1.69	0.000	16	20.0	252	10.8	1.94	0.024	0.18	0.671

***Source:*** Prepared by the authors from the study data.

**TABLE 4.** Low socioeconomic status, deprived geographic area, and other risk factors for cleft lip with or without cleft palate (adjusted by years and propensity scores), Argentina, 1992-2001

Risk factor	OR^a^	95% CI^b^		P
Low socioeconomic status	1.23	0.96	1.58	0.097
Deprived geographic area	1.00	0.75	1.35	0.983
Native ancestry	1.44	0.98	2.11	0.060
Maternal acute illness	1.45	1.12	1.88	0.005
Maternal chronic disease	1.19	0.86	1.63	0.290
Maternal medication	1.08	0.85	1.38	0.535
Prenatal visits < 5	1.68	1.33	2.12	< 0.001

***Source***: Prepared by the authors from the study data.

a OR = odds ratio.

b CI = confidence interval.

As expected, our results showed that populations with the most unfavorable social conditions resided in deprived geographic regions. Pregnant women in these areas had fewer prenatal control visits and more acute illnesses, and the women were also more often of native ancestry. Given the recognized high prevalence of CL±P in Amerindian populations (16), this last observation could explain the high frequency of CL±P we found in clusters of deprived areas.

No difference between ORs for low SES and the rest of the risk factors was observed in deprived areas versus not-deprived areas, indicating absence of interaction between risk factors and area of residence.

Propensity scores were used in the final model to evaluate the residual effects of unmeasured variables. An increased risk for CL±P observed in low-SES women (OR = 1.23) could not be explained by any single variable incorporated in the SES index. The slightly greater risk found might be due to residual effects of other factors correlated with low SES, such as environmental conditions (24 26), smoking (7, 27), alcohol (6), and malnutrition.

A number of similar studies have been performed, with differing results. Carmichael et al. (28) found no risk of individual SES with oral clefts. In a population-based study, Ericson et al. (29) observed the greatest risk of CL±P for subjects with the lowest SES, based on a multilevel index that combined individual and neighborhood measures. Yang et al. (30) showed consistently increased risks of selected birth defects associated with a low household SES index, but not with individual SES measures.

Whether or not the effects on health due to geographical deprivation and individual socioeconomic status are independent is still a controversial issue in the scientific literature (31). In general, successive adjustments for individual SES progressively reduce the magnitude of the association between GA deprivation and health (32). This shows that variables associated with low SES, at the individual level, are more relevant to adverse reproductive health than are variables related to the place of residence. It is unclear if there is an actual independent neighborhood effect or if an incomplete adjustment of individual SES is responsible for the small residual differences between residential areas (33).

Among all the variables we analyzed in our study, a reduced number of prenatal visits was the most important risk factor for CL±P. In addition, we observed a higher proportion of mothers with few prenatal visits in deprived GAs than in other areas.

There is a well-known association between poor prenatal care and such adverse reproductive outcomes as low birthweight, prematurity, and increased mortality (34). A study of newborns in South America and in the United States showed that adequate prenatal care was associated with larger birthweight increases in infants with CL±P than in healthy newborns (35). Given that low birthweight is a well-recognized comorbidity of CL±P, prenatal care for at-risk pregnancies is relevant to reducing the health burden of CL±P.

### Strengths and limitations

Our study sample included all of the cases and the controls born in 39 ECLAMC hospitals in Argentina, so a selection bias was unlikely.

Since the data on the maternal home address were unspecified in more than 50% of the sample, we used the hospital of birth as a proxy for maternal residence during pregnancy. This could have introduced bias in the exposure estimates, but we assume that most of cases were born in a hospital within a short distance of the maternal residence. This could have caused some bias with respect to the referral of cases with a prenatal diagnosis to tertiary centers outside the department of residence. However, since such a prenatal diagnosis for isolated CL±P is relatively rare, any such referral bias should have been modest.

In the ECLAMC work, the descriptions of congenital anomalies are reviewed by expert geneticists. The personal interviews with mothers are conducted by a qualified and experienced team. Before beginning to do data collection, all ECLAMC-affiliated professionals receive the same standard training from ECLAMC coordinators. Annual ECLAMC meetings are held, where further training is provided as needed. As a result, data quality and consistency are thought to be high. Nevertheless, maternal memory is an important caveat in case-control studies, and it is well known that exposure factors are more often reported for malformed newborns than for healthy ones. Therefore, despite the ECLAMC training, this type of bias cannot be discarded.

There is socioeconomic diversity among the areas where the ECLAMC hospitals included in this study are located. This makes it possible to compare regions with different deprivation levels and thus evaluate the impact of access to health services and of infrastructure quality.

Finally, unrecognized factors or unmeasured variables, due to unavailable data, could have affected our results. To control these potential biases, we applied a propensity score, which balanced the demographic characteristics of the cases and controls in our study.

### Conclusions

This study showed that, after adjusting for confounders, low SES is a slight risk factor for CL±P, independent of the geographical deprivation level. No interaction between individual SES and deprived GA was found. The number of prenatal visits differed across geographical deprivations levels, probably indicating limited access to health services in more deprived regions.

These results could be a guide for public health policies, indicating that factors related to low individual SES, such as poor education and prenatal control, as well as lifestyle factors, such as smoking and alcohol, should be primarily targeted as risk factors for CL±P, rather than those related to a deprived place of residence.

## Acknowledgments.

The authors wish to thank all the people working cooperatively in ECLAMC, a network that has been active for more than 40 years.

## Funding.

Support came from the Consejo Nacional de Investigaciones Científicas y Técnicas (CONICET) of Argentina and from the Scientific Research Commission of Buenos Aires (CICPBA).

## Disclaimer.

Authors hold sole responsibility for the views expressed in the manuscript, which may not necessarily reflect the opinion or policy of the *RPSP/PAJPH* or PAHO.

## Appendix

**APPENDIX I.** Annual births in ECLAMC hospitals and percentage of total birth population by department/*partido* in Argentina

Department/*Partido*	Province	Annual births in ECLAMC hospitals 2001	Total births in department/*partido* in 2001	% of total births in ECLAMC hospitals
CABA	Bs As	13 411	42 375	31.65%
Almirante Brown	Bs As	2 807	9 199	30.51%
Lomas de Zamora	Bs As	1 000	10 088	9.91%
Esteban Echeverría	Bs As	1 878	4 406	42.62%
General San Martin	Bs As	600	6 120	9.80%
Lanús	Bs As	1 609	6 461	24.90%
Avellaneda	Bs As	590	5 691	10.37%
General Pueyrredón	Bs As	4 702	9 675	48.60%
La Plata	Bs As	1 311	10 711	12.24%
Gualeguaychu	Entre Ríos	1 045	1 824	57.29%
Paraná	Entre Ríos	3 945	5 706	69.14%
Rosario	Santa Fe	5 631	9 757	57.71%
Capital	Córdoba	6 053	20 517	29.50%
San Martin	Mendoza	2 057	2 152	95.59%
Capital	Mendoza	1 886	2 002	94.21%
Guaymallen	Mendoza	550	4 861	11.31%
Capital	San Luis	2 677	3 757	71.25%
Posadas	Misiones	4 689	6 533	71.77%
San Miguel de Tucumán	Tucumán	10 090	10 372	97.28%
Dr. Manuel Belgrano	Jujuy	2 502	4 919	50.86%
Capital	La Rioja	1 730	3 174	54.51%
Futaleufu	Chubut	1 500	806	186.10%
Bariloche	Rio Negro	306	2 075	14.75%
Biedma	Chubut	529	1 270	41.65%
Ushuaia	T. del Fuego	230	956	24.06%

**APPENDIX II.** Propensity scores, which form five strata where socioeconomic variables are similar for cases and controls in each of them; the propensity scores were used to adjust the multivariate logistic analysis

Strata		Maternal age^a^	Gravidity^b^	Maternal education^c^	Paternal education^c^	Paternal occupation^e^
		Mean	Mean	Median	Median	Median
**1**	Controls	27.9	3.6	4	4	4
	Cases	27.8	3.9	4	4	4
**2**	Controls	25.0	2.7	5	5	5
	Cases	25.2	2.4	5	5	5
**3**	Controls	24.1	2.7	5	5	5
	Cases	24.3	2.8	4	4	4
**4**	Controls	22.9	2.8	4	4	3
	Cases	22.7	3.1	4	4	3
**5**	Controls	22.6	3.0	4	4	3
	Cases	22.2	3.3	4	4	3

a Maternal age (in years).

b Gravidity (number of pregnancies).

c Maternal education and paternal education in 8 categories: 1- no schooling, not read, 2- no schooling, only read, 3- incomplete grammar school, 4- complete grammar school, 5- incomplete secondary school, 6- complete secondary school, 7- incomplete university, 8- complete university. (Maternal and paternal education < 5 was considered low education.)

d Paternal occupation: 1-unemployed, 2- househusband, 3- odd job/unskilled labor, 4- skilled labor, 5-independent labor, 6- manager, 7- clerk (white collar), 8- professional/university. (Paternal occupation < 5 was considered low occupation.)
